# Metformin effects on zonulin level in polycystic ovarian women

**DOI:** 10.5599/admet.905

**Published:** 2020-12-09

**Authors:** Manal Ibrahim, Mutaz Ahmeid

**Affiliations:** 1 Master of Clinical Biochemistry, College of Pharmacy /University of Mosul, Iraq; 2 Ph.D. Clinical Biochemistry, College of Medicine /University of Tikrit, Iraq

**Keywords:** HOMA-IR, Homeostatic Model Assessment for Insulin Resistance, PCOS, Polycystic Ovarian Syndrome, FSH, Follicle Stimulating Hormone, LH, Luteinizing Hormone, SHBG, Sex Hormone Binding Globulin

## Abstract

Zonulin protein is a haptoglobin precursor and functions to modulate the permeability of tight junctions between enterocytes. Local inflammation or systemic inflammation can trigger zonulin expression. While the increased zonulin level causes an increase of intestinal permeability and entrance of foreign antigens, the latter can increase insulin resistance and inflammation. Polycystic ovarian syndrome affects women during their reproductive age characterized by hyperinsulinemia and/or hyperandrogenemia and associated with infertility problems. Changes in gut permeability such as irritable bowel syndrome are often found in PCOS patients. While metformin increases insulin mediates glucose uptake and, acts as an insulin-sensitizing drug used to treat PCOS patients is recently discovered to reshape intestinal bacteria and hence may affect intestinal action. This study was designed to find any association between zonulin level and other parameters in PCOS patients and to find metformin treatment effects on zonulin in PCOS patients. Thirty-one newly diagnosed PCOS women agree to take metformin 850 mg twice daily for three months and, and to give fasting serum samples to measure zonulin, FSH, LH, total testosterone, free testosterone, SHBG, fasting insulin, and fasting serum glucose before and after treatment. The free testosterone and zonulin are determined by the ELISA technique while the other parameters are determined by the Cobas technique. According to patients’ Homeostatic Model Assessment (HOMA-IR), the Patients were divided into two sub-groups: the first group consisting of those with initial HOMA-IR less than two and the second group was those of an initial HOMA-IR of between two to four. Whereas the first group showed no significant response to treatment, the second group showed a better response to metformin treatment, as demonstrated by their LH, total testosterone, free testosterone, fasting insulin, zonulin, and glucose levels. These parameters markedly improved after metformin treatment with p-values of 0.08, 0.09, 0.07. 0.04, 0.01 and 0.06, respectively.

## Introduction

The polycystic ovarian syndrome can affect women during their reproductive age at an incidence rate of 3-10 % and characterized by sex hormone imbalance [1,2]. Irregular menstrual cycle, polycystic ovary morphology, and hirsutism are the most frequent symptoms, with features of infertility and insulin resistance [3,4]. Insulin resistance and obesity play roles in PCOS pathophysiology [5,6]. Insulin resistance gives rise to hyperinsulinemia [7], which directs ovarian steroidogenic enzymes to produce more androgen and abolish ovulation [8,9]. Therefore, insulin-resistant PCOS patients show more metabolic lipogenic changes, and abnormal steroidogenesis because of a decrease in insulin signaling, while non-insulin resistant PCOS patients show abnormality in steroidogenesis only [10,11]. The HOMA-IR is a technique used to assess the β-cell function and insulin resistance from basal glucose and insulin and it equalizes ((Fasting Insulin multiplied by Fasting glucose) divided by 22.5), normal HOMA IR is 1 [12,13]. Insulin resistance occurs when fasting insulin is HOMA-IR>2 [14].

Zonulin is a haptoglobin precursor that has a molecular-weight of 47 kDa and modulates the permeability of tight junction (TJ) between cells of the digestive tract [15,16]. An increase in zonulin is associated with an increase in gut permeability. Zonulin expression is triggered by either local inflammation or systemic sub-clinical inflammation [17] and is suggested to have a role in the production of metabolic syndrome and insulin resistance [18]. In PCOS women, zonulin is correlated with insulin resistance and the severity of the menstrual disorder [19]. Moreover, irritable bowel syndrome and chronic fatigue syndrome, two conditions common in PCOS are linked to an increase in gut permeability [20,21]. Metformin drug used in PCOS treatment can decrease hepatic gluconeogenesis by different mechanisms [22]: delay intestinal glucose absorption [23], reshape microbiota in the intestine [24,25], increase insulin-mediated glucose uptake in the liver, visceral fat, and muscle [26], and stimulates insulin signaling pathway in human granulosa cells and other cells of the body [27]. This insulin-sensitizing drug, promises PCOS patients, may play a role in zonulin level and gut permeability. Therefore, the study is designed to find the metformin effects on zonulin level in PCOS patients and its association with other parameters.

## Experimental

Thirty-one newly diagnosed PCOS women according to Rotterdam criteria are included in this study. Patients with adrenal dysfunction, thyroid dysfunction, diabetics, and those on oral contraceptives are excluded from the study. Fasting insulin, fasting glucose, FSH, LH, testosterone, free testosterone, SHBG, and serum zonulin are measured before and after intake of metformin (Bigomet®), 850 mg twice daily for three months. The research plan was approved by the Scientific Committee of the College of Medicine, Tikrit University, on 24/October/ 2018. The parameters of FSH, LH, total testosterone, SHBG, and insulin are determined by Cobas technique (Cobas e 411 systems); fasting serum glucose was determined by Cobas c 311analyzers. Serum zonulin has been determined according to “Human zonulin ELISA kit Cat NO. MBS2607261 “supplied by Mybiosource/USA Company. Free testosterone is determined by the ELISA technique. Statistical analysis performed by SPSS and the significant changes was considered when p-value less 0.05. The patients are divided into two subgroups according to their initial Homeostatic Model Assessment (HOMA-IR) that is determined before starting metformin treatment.

### First group

Sixteen patients have initial HOMA-IR less than two, eleven of them have primary infertility and only five women have secondary infertility, and their mean age was 28.81 years.

### Second group

Fifteen patients have initial HOMA-IR two to four, twelve of them have primary infertility and only three of them have secondary infertility, and their mean age was 26.93 years.

## Results and discussion

First group, those of initial HOMA-IR <2, did not show any significant difference in any of the parameters that measured including BMI, FSH, LH, testosterone, free testosterone, SHBG, fasting insulin, fasting glucose, HOMA IR, and zonulin as seen in Table 1.

The second group those of initial HOMA-IR 2-4 (insulin resistance group), showed better response to metformin treatment (Table 2). This group exhibits a marked decrease in LH, total testosterone, free testosterone, fasting glucose with a p-value of 0.08, 0.09, 0.07 and 0.06 respectively, and a significant decrease in fasting insulin, HOMA-IR, and zonulin with p-values of 0.04, 0.02 and 0.01 respectively.

The study showed no change in body mass index (BMI) (Tables 1 and 2), which is in agreement with Sharma et al 2019 study [28]. It is well known that most PCOS women are obese, and they have an increase in blood volume and the volume of distribution of any drug [29]. Therefore, a higher dose of any drug is required to do equivalent action in non-obese patients [30].

The first group, those of initial HOMA-IR less than two (Table 1), exhibited no significant decrease in their HOMA-IR and fasting glucose after metformin treatment, while the second group, those of initial HOMA-IR two to four (Table 2) exhibited a significant decrease in HOMA-IR after the end of three months therapy with a p-value of 0.04, and a marked decrease in fasting glucose, with a P-value of 0.06. This result agrees with the study of Nawrocka and Starczewski, who showed that metformin can significantly decrease HOMA-IR only in insulin-resistant PCOS patients [31]. Moreover, the first group (Table 1) showed no significant changes in zonulin value after treatment, while the second group (Table 2), showed a significant decrease in zonulin with a P-value of 0.01, suggesting that metformin can decrease zonulin in insulin-resistant patients with no menstrual disturbances. Ilhan and Yildizhan study suggested that zonulin is a pathogenic novel biomarker in PCOS women and there is a strong correlation between HOMA-IR and zonulin in PCOS patients [32]. Also, Zhang et al suggested that zonulin plays a role in PCOS pathogenesis and can be used as a marker for detection of the severity of disease and in the prognosis of PCOS treatment [19]. This is obvious, especially that increase zonulin leads to alteration in gut permeability, decrease the integrity of the intestinal barrier allowing an excess of the infectious agent and dietary antigen to facilitate mucosal immune elements such as IL-6 which can lead to insulin resistance [33]. We proposed that the metformin effect on zonulin is related to the antibiotic effect of metformin on the gut and modulation of intestinal microbiota. However, the first group of those of initial (HOMA-IR <2) exhibited no changes in zonulin value as there is no difference in gut permeability and microbiota composition before and after treatment, as non-insulin resistance associated with no alteration in intestinal permeability. This agrees to a certain degree with Cetin’s study conclusion, who included non-obese, normal HOMA-IR PCOS patients in his study and suggested that insulin resistance is not triggered if there is integrity in the gut permeability [34]. It seems that this study is the first study to show the effect of metformin on zonulin in PCOS patients. We found that metformin can decrease zonulin in insulin-resistant PCOS patients with no menstrual irregularities and not prone to have diabetes, and we suggest that metformin effects on zonulin are not related to insulin-sensitizing activity but may be due to the modulation of intestinal bacteria.

Testosterone and free testosterone showed no significant decrease in the non-insulin resistant group (Table 1) p-value 0.13 and 0.12 respectively, while the second group showed a marked but insignificant decrease in total testosterone p-value 0.09 and free testosterone p-value 0.07. This result agrees with Baqer et al study in 2018 when they administer metformin 500 mg three times for three months and showed an insignificant decrease in total testosterone [35]. Metformin can suppress androgen production by its action on the ovary and adrenal gland and increase SHBG production by the liver [23]. Different studies revealed that metformin causes a reduction in androgen, an increase of SHBG, and a restoration of the normal menstrual cycle [36-40] but this study reveals a slight insignificant increase in SHBG in both groups, which agree with Singh *et al.* study who show no significant decrease in serum testosterone, fasting plasma glucose and BMI with a significant decrease in HOMA-IR, after intake of metformin 500 mg twice daily for 3 months [41]. On the other hand, Upadhyaya and Rehan study has reported that women of PCOS with hyperinsulinemia show a significant reduction in testosterone in compare to the normo-insulinemic group after metformin treatment and they suggested that hyperinsulinemic, normo-androgenic PCOS is a favorable condition for metformin response [42], also Moghetti et al reveal that metformin responders usually had higher fasting plasma insulin [43]. Concerning the level of LH, the second group (Table 2) showed a decrease in LH with a p-value of 0.08, while the first group of less insulin-resistant patients (Table 1) exhibited a p-value of 0.25. This is attributed to that the hyperinsulinemia increased androgen production by augmenting the LH effect for androgen production, while metformin can reverse this by decrease insulin level [44].

## Conclusions

Metformin alone does not affect body mass index within three months of treatment and has no effect on insulin level in non-insulin resistant PCOS patients, but can decrease insulin in insulin-resistant PCOS patients. Concerning zonulin, the significant decrease in zonulin in women with higher insulin resistance may be attributed to the metformin effect on intestinal bacterial distribution. These results give rise to conclude that patients with higher HOMA-IR value before treatment (insulin resistant) are better metformin responders.

## Figures and Tables

**Table 1.** Statistical analysis of parameters and zonulin before metformin therapy in PCOS with initial (before treatment) HOMA-IR <2

Initial HOMA-IR<2		Mean +sd	p-value
BMI	Before	28.26+5.93	0.4
	After	26.6+4.13	
FSH(uIU/mL)	Before	6.16+1.69	0.28
	After	7.66+4.15	
LH(uIU/mL)	Before	10.26+5.04	0.25
	After	7.83+5.47	
Testosterone(ng/mL)	Before	0.55+0.4	0.13
	After	0.36+0.26	
free testosterone (pg/mL)	Before	2.86+0.74	0.12
	After	2.4+0.72	
SHBG (nmol/L)	Before	34.01+33.82	0.9
	After	35.26+15.12	
Fasting Insulin (uIU/mL)	Before	4.29+2.72	0.15
	After	9.13+10.11	
Fasting glucose (mg/dL)	Before	89.63+11.86	0.27
	After	95.33+13.66	
HOMA-IR	Before	0.96+0.62	0.13
	After	2.43+2.93	
Serum zonulin (ng/mL)	Before	20.83+15.46	0.52
	After	16.90+15.21	

**Table 2.** Statistical analysis of parameters and zonulin before and after metformin therapy in PCOS with initial (before treatment) HOMA-IR = 2-4

Initial HOMA-IR=2 – 4		Mean +SD	p-value
BMI	Before	28.95+8.55	0.3
	After	27.04+4.48	
FSH (uIU/mL)	Before	5.94+2.39	0.11
	After	4.68+1.89	
LH (uIU/mL)	Before	12.11+8.08	0.08
	After	7.24+6.01	
Testosterone(ng/mL)	Before	0.65+0.48	0.09
	After	0.39+0.28	
free testosterone (pg/mL)	Before	2.84+0.92	0.07
	After	2.38+0.6	
SHBG (nmol/L)	Before	27.27+18.04	0.2
	After	38.12+22.64	
Fasting Insulin(uIU/mL)	Before	12.74+4.29	0.04*
	After	8.7+5.33	
Fasting glucose (mg/dL)	Before	89.73+24.2	0.06
	After	82.91+8.37	
HOMA-IR	Before	2.8+0.93	0.02*
	After	1.79+1.17	
Serum zonulin (ng/mL)	Before	16.26+10.10	0.01*
	After	7.92+5.09	

*p-value less than 0.05
